# Cross sections of $^{147-149}$Sm($^{6}$Li,x) reactions for the production of $^{149}$Tb for targeted alpha therapy

**DOI:** 10.1038/s41598-025-24894-9

**Published:** 2025-11-20

**Authors:** Laura A. Bills, Alan B. McIntosh, Jonathan T. Morrell, Philip Adsley, Austin D. Abbott, Diana Carrasco Rojas, Jeremias Garcia-Duarte, Matthew D. Gott, Kris Hagel, Travis Hankins, Jason T. Harke, Bryan M. Harvey, Richard O. Hughes, Lauren A. McIntosh, Yonatan Mishnayot, Connor Mohs, Gabriela A. Picayo, Madison Reuter, Robert Rider, John Santucci, Sophia Sauceda, Maxwell Sorensen, Alexandra Tabacaru, Aaron S. Tamashiro, Evgeny E. Tereshatov, David Thomas, Zachary Tobin, C. Etienne Vermeulen, Benjamin Wellons, Sherry J. Yennello

**Affiliations:** 1 Cyclotron Institute, Texas A&M University, College Station, Texas 77843 USA; 2 Department of Chemistry, Texas A&M University, College Station, Texas 77843 USA; 3 Los Alamos National Laboratory, Los Alamos, New Mexico 87545 USA; 4 Brookhaven National Laboratory, Upton, New York 11973 USA; 5 Department of Physics & Astronomy, Texas A&M University, College Station, Texas 77843 USA; 6 Department of Physics, University of Texas at El Paso, El Paso, Texas 79968 USA; 7 Lawrence Livermore National Laboratory, Livermore, California 94550 USA; 8 Physics Division, Argonne National Laboratory, Lemont, Illinois 60439 USA; 9 Department of Physics, Texas Lutheran University, Seguin, Texas 78155 USA; 10 Present Address: Enrichment Science and Engineering Division, Oak Ridge National Laboratory, 37830 Oak Ridge, TN USA; 11 Present Address: Soreq Nuclear Research Center, 818000 Yavne, Israel

**Keywords:** Cancer, Chemistry, Physics

## Abstract

Terbium-149g ($t_{1/2}$ = 4.12 h) is of particular interest for targeted alpha therapy cancer treatment due to its ability to decay via both alpha and positron emission, making it a potential theranostic nuclide. Due to many challenges facing its production, there are limited facilities worldwide that have demonstrated the ability to produce this nuclide in quantities sufficient for medical research. Since the Cyclotron Institute at Texas A&M University is a specialized accelerator facility capable of accelerating a wide variety of ions, we are investigating production pathway options. One of the major challenges facing its production is the known co-production of the excited isomeric state, $^{149\textrm{m}}$Tb ($t_{1/2}$ = 4.1 min). However, this state does not decay to the ground state of $^{149\textrm{g}}$Tb, negating any potential contribution to its yield. Due to its short-half life, the cross section for the population of this state has never been measured. After calculating several potential reaction yields using predictive models, the reactions of $^{147-149}$Sm($^{6}$Li,xn)$^{149}$Tb were identified as candidates. Lithium-6 beams of varied energies between 45-65 MeV were impinged on enriched $^{147}$Sm, $^{148}$Sm, and $^{149}$Sm targets at the Cyclotron Institute at Texas A&M University, and the reaction products were measured immediately following irradiation using high-purity germanium detectors, enabling detection of both $^{149\textrm{m}}$Tb and $^{149\textrm{g}}$Tb. Cross sections for all nuclides produced in sufficient activity in these reactions were also measured and reported here. We conclude that the population of $^{149\textrm{m}}$Tb is much preferred over population of the ground state for these $^{6}$Li-induced reactions, and it is necessary to explore other options for $^{149\textrm{g}}$Tb production.

## Introduction

Targeted alpha therapy (TAT) is an emerging field of cancer therapy research that has the advantage of targeting and damaging cancer cells with minimal damage to surrounding healthy tissues. This is accomplished through the binding of an alpha-emitting radionuclide to a biomolecule that binds to cancer cells. Once the biomolecule transports the radionuclide to the tumor site, it decays, depositing most of its energy directly into the tumor, therefore minimizing the damage to surrounding healthy cells ^1^. This method is potentially advantageous compared to alternative methods of radiation cancer therapy, as some of these other methods utilize longer penetration distances through the body and have the potential to cause damage to larger amounts of healthy tissue in addition to the cancerous cells.

Ideal candidates for TAT are often nuclides with short half-lives that decay quickly within the body; there is little long-term radiation dose. In addition, both the nuclides and their decay products should be chemically nontoxic. The decay products should also be stable or sufficiently long-lived to remain harmless to the biological system in the small amounts utilized for TAT. Using these criteria, only a few radionuclides have been identified as possible TAT nuclides ^2–4^. Since these nuclides are radioactive with relatively short-half lives, they must be synthetically produced, which is often challenging.

Of the identified TAT radionuclides, $^{149}$Tb is particularly attractive in that it is the only one that decays via both positron and alpha particle emission, potentially allowing it to be imaged by positron emission tomography (PET), while simultaneously treating the cancerous cells ^2^. As the other identified nuclides do not decay by positron emission, they can not be used for PET, though for some SPECT may be possible. This eliminates the need for the co-development of a separate imaging analog and enables both imaging and therapy with a single nuclide.

$^{149g}$Tb is produced with particle accelerators, due to its significant deviation from the line of stability. Although this nuclide has been produced through a variety of different heavy-ion induced reactions ^5–8^, its main methods of production to date are through high-energy proton spallation on a tantalum target ^9,10^ and through the $^{151}$Eu($^{3}$He,5n)$^{149}$Tb reaction ^11–13^. Since these production mechanisms require unique accelerator facilities, there are very few sites globally with the ability to produce $^{149}$Tb, making it important to explore other production pathways to increase the availability of this nuclide. Several other experiments have shown $^{149}$Tb yields in the low 100s of mb through the irradiation of enriched $^{152}$Gd targets ^14,15^. However, given the low natural abundance of $^{152}$Gd (0.2%), these targets are not ideal for routine production; the cost for such rare nuclides is high ^16^. Many of these previous experiments also demonstrate the production of the metastable isomer $^{149m}$Tb (t$_{1/2}$ = 4.1 min), but no cross sections have been reported due to its short half-life ^6,7,17^. Given the high-spin state of this isomer, it preferentially decays to $^{149}$Gd, instead of the ground state of $^{149}$Tb. Therefore, the production of the $^{149m}$Tb isomer ultimately hinders the yield of $^{149g}$Tb, the alpha-emitting radionuclide necessary for the therapy component of TAT.

In this work, we measure the production cross sections of $^{149\textrm{g}}$Tb and $^{149\textrm{m}}$Tb using $^{6}$Li beams of various energies on Sm targets. The reaction cross sections predicted by the reaction codes of PACE4 ^18,19^, ALICE 2017 ^20^, and EMPIRE ^21^ were used to identify Li as a promising projectile, due to predicted cross sections for $^{149}$Tb in the hundreds of millibarns. These reaction codes were then used to guide the selection of the beam energies that were predicted to maximize the production of $^{149}$Tb. Previous experiments have been performed by irradiating samarium targets with a lithium beam ^22–25^; however, these experiments were carried out at lower energies using different isotopes of samarium than those used in the present work. Since samarium has relatively low mass stable isotopes compared to other lanthanides in the region, $^{147-149}$Sm were chosen to reduce the projectile energy, reducing the spin of the entrance channel, therefore populating more of the ground state.

The reactions measured in this study are $^{147}$Sm($^{6}$Li,x) at 45 MeV, $^{148}$Sm($^{6}$Li,x) at 55 MeV, and $^{149}$Sm($^{6}$Li,x) at 55, 60, and 65 MeV to determine partial excitation functions for these systems.

## Methods

### Target preparation

Aluminum target frames were fabricated using aluminum 6061 alloy. Metallic aluminum foils with quoted thicknesses of approximately 800 µg/cm$^{2}$ were adhered to the frames using Instant Adhesive 910 Metal Bonding General Purpose (Permabond Engineering Adhesives, Batch BK0042). The thickness of the foils was then measured by placing the foil over an aluminum collimator between a californium-249 source and a silicon detector (ORTEC, model no. BU-019-300AS). The loss in energy of the alpha particles through the foil was measured by the silicon detector and analyzed using Genie$^{\textrm{TM}}$ 2000 (Mirion Technologies, Inc.) to calculate the thickness of the aluminum backing foils. Enriched, metallic samarium foils were fabricated from enriched samarium oxide powders acquired from Oak Ridge National Laboratory (enrichment levels, $^{147}$Sm: 97.8%, $^{148}$Sm: 96.4%, and $^{149}$Sm: 97%). Since the enriched material comes in the oxide form for stability purposes, the oxide needed to be reduced *in situ* using zirconium metal. A pellet of 181 mg of Sm$_{2}$O$_{3}$ and 108 mg of Zr metal shavings was pressed using a die and placed inside a tantalum pinhole boat (S17B, RD Mathis Company). The boat was then mechanically sealed and placed inside an evaporator ^26^. The aluminum backing foils were placed 7 cm above the pinhole boat and the evaporation was monitored via a 6 MHz quartz crystal monitor (QI8010, Fil-Tech, Inc.) to measure the thickness, and a thermocouple was placed on the backing foil to monitor the temperature. From previous tests using natural material, it was determined that the evaporation should be paused when the substrate temperature reached $60^\circ$C, as higher temperatures resulted in oxidation of the foil. This effect was hypothesized to be related to the decomposition of the adhesive. The evaporation was continued once the foils had cooled, until a thickness of about 1 mg/cm$^{2}$ was achieved. The areal density of the resulting targets was then measured via alpha loss using the same setup. The targets were then stored under vacuum until irradiation to prevent oxidation. All targets and backings measured via alpha loss were assumed to have a 10% systematic error introduced by the measurement of the alpha energy loss through the targets. The areal density of 1 mg/cm$^{2}$ was chosen as it did not cause significant energy loss of the beam through the target, allowing for the measurement of a cross section at a specific energy.

### Experimental setup

Lithium beams were accelerated using the K150 Cyclotron at TAMU. Four modules of the Hyperion detector array surrounded a thin-walled aluminum vacuum chamber containing the target during irradiation. The Hyperion detectors are composed of a bismuth germanate (BGO) Compton shield surrounding a HPGe clover each containing four individual HPGe crystals ^27^. Since this experiment was designed to measure nuclides with relatively high cross sections (on the order of millibarns) by monitoring their decay post-irradiation, only 4 of the possible 14 detectors were used. These detectors were placed at angles of $90^\circ$ and $135^\circ$ to the left and right of the beam so that none of the detectors were downstream of the target to avoid unnecessary neutron damage during the irradiations. Each individual HPGe crystal was connected to a high-voltage power supply (HVPS) (EHS 82 60p, iseg Spezialelektronik GmbH), preamplifier power supply (ORTEC 4003 Preamplifier Power Output), a bias shutdown designed to turn off the detector if it was not cooled properly, and a signal output connected to a digitizer module (MDPP-16, Mesytec). All BGOs in a module were biased with a common voltage supply; all BGO anodes in a module were connected in series and sent to a single digitizer (MDPP-16, Mesytec). All signals from the HPGe detectors were digitized using the mvme - mesytec VME Data Acquisition software (mesytec GmbH) ^28^. A more detailed explication of the experimental setup is described in Reference 29. ^29^

The targets were visually monitored for degradation during the irradiation using a small camera inside the target chamber. The beam intensity was continuously monitored via an unsuppressed Faraday cup located in the beam dump, which was later calibrated against an electron-suppressed Faraday cup at the target location.

Overnight irradiations between 10-12 hours were chosen to prevent excess accumulation of longer lived isotopes, such as $^{151}$Tb (t$_{1/2}$ = 17.6 h) and $^{148}$Eu (t$_{1/2}$ = 54.4 d). This design also allowed for a new beam energy to be tuned during the day in preparation for the next target irradiation at night. The targets were measured during and immediately after the irradiation to measure the decay of short-lived radionuclides. Following a few hours of counting, the foils were then removed from the Hyperion array and transferred to one of two single-crystal HPGe detectors (Canberra Model No. GC2020 & Canberra Model No. BE2020), to further measure the long-lived nuclides that were produced and to prepare the Hyperion chamber for the next irradiation. The foils were counted in an inert N$_{2}$ environment, with a thin layer of Kapton tape between the crystal and the foil to minimize oxidation during these longer counting periods. The single-crystal HPGe detectors collected data using Genie$^{\textrm{TM}}$ 2000.

### Detector calibrations

The python package Curie ^30^ was used to perform the calibration, peak fitting, and resulting cross section calculations. The ROOT Data Analysis Framework ^31^ was also utilized for some of the data analysis. Each detector was calibrated for energy, efficiency, and resolution using a $^{152}$Eu source (BC-6311, Eckert & Ziegler Nuclitec GmbH) to cover a wide range of energies. For the Hyperion detector array, the source was mounted to the target ladder in the target position located about 8 cm from the detector faces, separated by the aluminum wall of the target chamber. Curie was then used to calculate the individual detector leaf efficiencies based on the provided source spectrum and known activity ^30^. The background under each peak was estimated using the SNIP algorithm ^30,32^. All of the energies and branching ratios for these fits were referenced from the NNDC NuDat database, with all associated uncertainties ^33^. The efficiency calibrations of each individual HPGe detector (leaf) of Hyperion were found to be in close agreement with each other and consistent with the predicted efficiencies calculated using GEANT4 by Hughes et al. ^27^. Once the individual leaves were calibrated, they were summed together using their respective energy calibrations, resulting in a total Hyperion detector array efficiency. This total efficiency also agrees with the GEANT4 model for the given number of clovers that were used ^27^.

For the single-crystal detectors, the same $^{152}$Eu source was used and was counted inside of the N$_{2}$ sealed box separated by a piece of Kapton tape to mimic the counting position of the irradiated foils. The efficiency curves for the entire Hyperion array and each of the single-crystal detectors are provided in the Supplementary Information.

### Peak fitting

**Figure 1:**
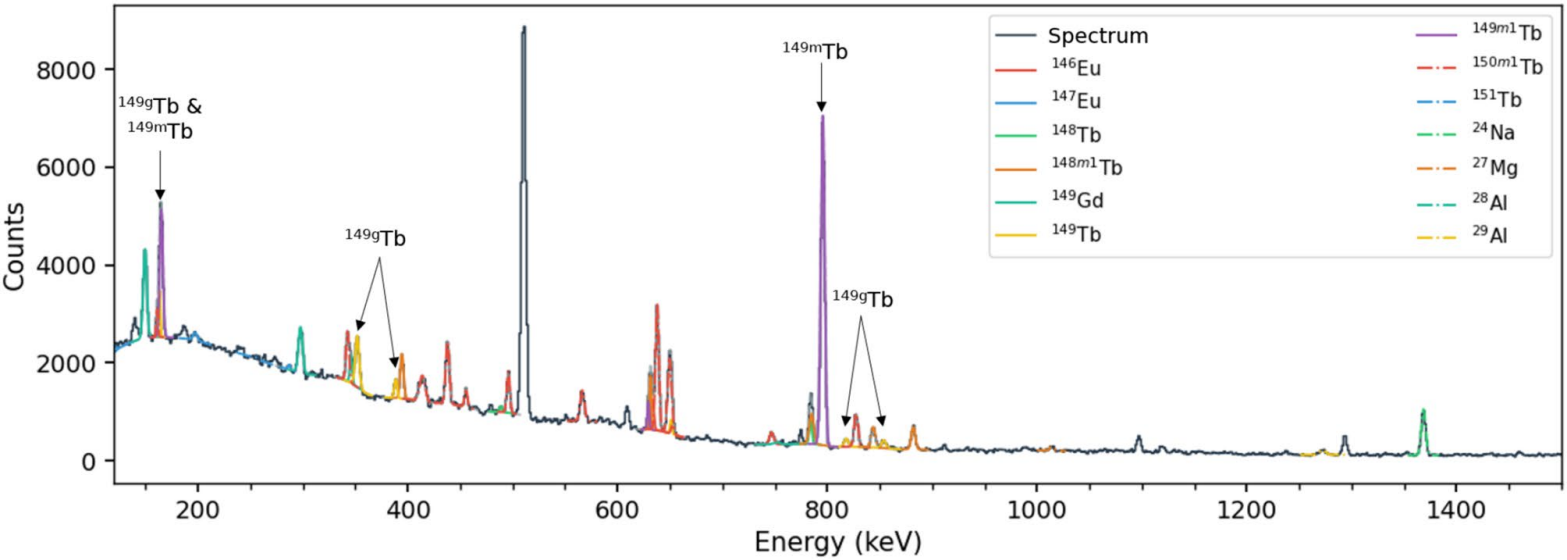
Gamma ray energy spectrum from 0 to 1500 keV measured with the Hyperion clovers for the first 30 minutes after irradiation of $^{147}$Sm with $^{6}$Li @ 45 MeV. The colored lines show fits to the data for known gamma rays from expected nuclides as indicated by the legend.

The calibrated $\gamma$-ray energy spectra were passed as inputs to the Curie python package, along with a list of isotopes thought to be present in each spectrum based on the produced isotopes as predicted by ALICE, PACE4, and EMPIRE. Using the NNDC database of $\gamma$-ray transition energies and branching ratios ^33^, a variety of nuclides for each experiment were correctly identified and fit. The peak fits were all performed using single or multiple Gaussian fits, depending on the number of peaks in the fitting region. In Fig. 1, the calibrated spectrum showing the number of measured counts at each energy in keV measured by the Hyperion detector array is shown. The spectrum was taken for a total of 30 minutes immediately after the irradiation of the $^{147}$Sm target. Curie was used to identify and fit all peaks, the accuracy of which is illustrated in Fig. 1. This figure also shows the large number of isotopes produced during the irradiations that can be simultaneously identified.

**Figure 2:**
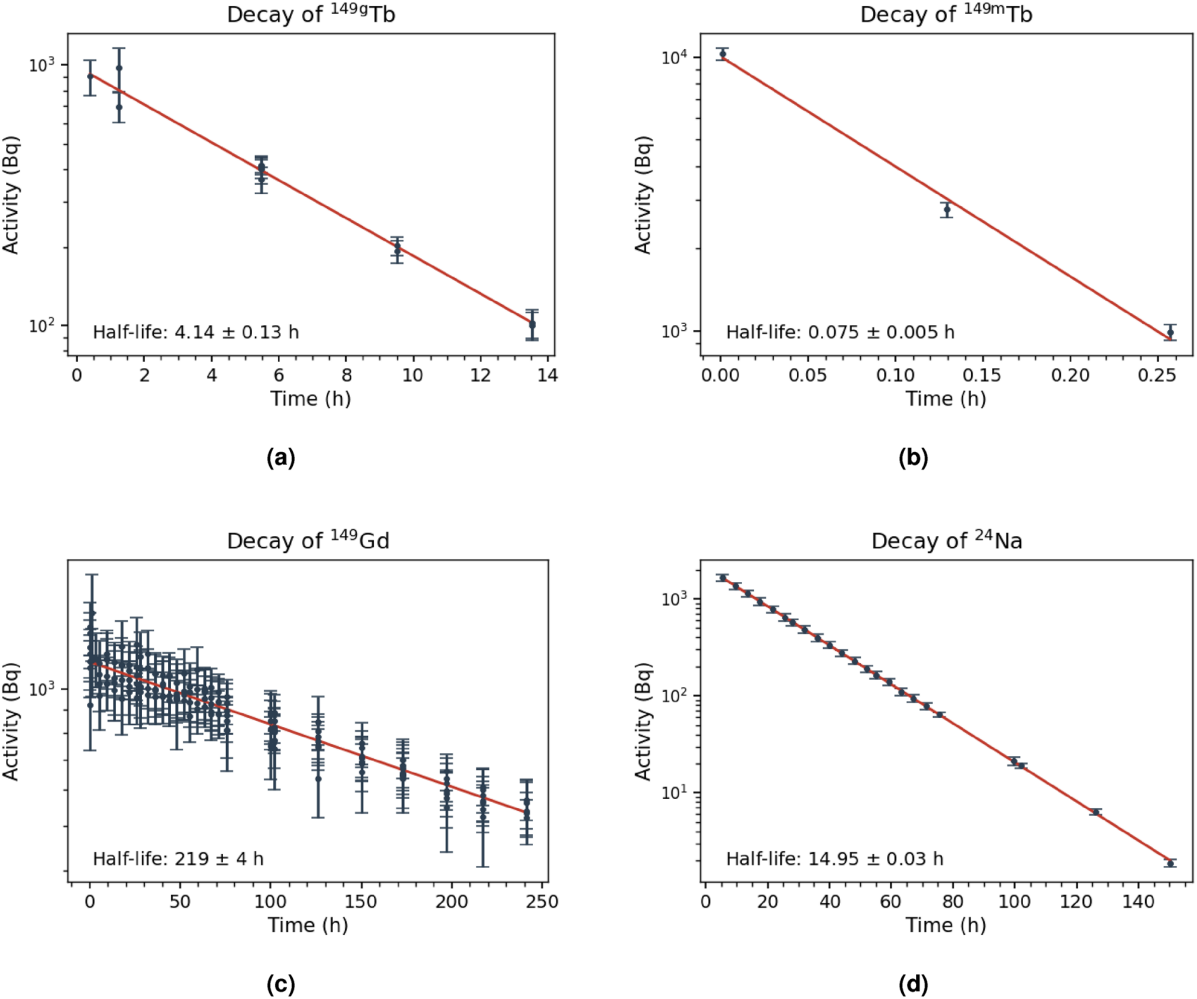
Decays of $^{149\textrm{g}}$Tb (t$_{1/2,\textrm{lit}}$ = 4.12 h; E$_{\gamma }$ = 464.85, 652.12, 861.86, 1341.19, 1640.26 keV; I$_{\gamma }$ = 0.0573, 0.165, 0.0760, 0.0233, 0.0322), $^{149\textrm{m}}$Tb (t$_{1/2,\textrm{lit}}$ = 0.07 h; E$_{\gamma }$ = 796.0 keV; I$_{\gamma }$ = 0.97), $^{149}$Gd (t$_{1/2,\textrm{lit}}$ = 222.7 h; E$_{\gamma }$ = 149.735, 214.277, 260.736, 272.321, 298.634, 404.296, 459.814, 534.295 keV; I$_{\gamma }$ = 0.48, 0.00195, 0.0130, 0.0322, 0.279, 0.00198, 0.0059, 0.0312), and $^{24}$Na (t$_{1/2,\textrm{lit}}$ = 14.96 h; E$_{\gamma }$ = 1368.626, 2754.007 keV; I$_{\gamma }$ = 0.99994, 0.99867) as a function of time after the end of irradiation of $^{149}$Sm with $^{6}$Li @ 60 MeV, where t = 0 represents the end of the irradiation.

In order to confirm the correct nuclide responsible for each of the fitted peaks, the areas under each peak were extracted and used to determine the activity of each nuclide at multiple time points following irradiation. Examples of the decay curves for selected nuclides with varying half-lives can be seen in Fig. 2, where the activities (in Bq) calculated from individual peak fits at different times are plotted on a logarithmic scale. For $^{149}$Tb and $^{149}$Gd, the activity was calculated from several different $\gamma$-ray transitions observed in the same spectrum. The resulting half-life calculated from the red logarithmic fit of the data is shown in the bottom left of each figure. When these half-lives are compared with the literature values, there is very good agreement, indicating that the $\gamma$-transitions were correctly identified with their respective radionuclide. When $\gamma$-rays from different nuclides were too close in energy to be resolved by the detectors, peak fitting became highly uncertain as the percentage of contribution from each nuclide was unable to be determined. To account for this, any fit with an error exceeding 25% of the total peak yield was omitted. The plots in Fig. 2 were also gated on singular $\gamma$-transitions to determine which energies were misidentified based on an incorrect measured half-life for the individual transition. This allowed for the omission of these energies, due to clear contamination from other transitions in the target or background. Finally, the 496.4 keV $\gamma$-ray from $^{149}$Gd was found to be impacted by coincidence summing from sequential decays and was therefore also excluded from the analysis and not shown in Fig. 2. After applying these criteria, the remaining decay curves yielded half-lives consistent with known literature values, therefore confirming that the utilized peak fits do correspond to their identified nuclide, and that each of these nuclides is present in the spectrum. Once the nuclides were confirmed based on their $\gamma$-transitions and half-lives, the half-lives were fixed using their literature values and uncertainties for the yield calculations.

### Cross section calculations

The beam current fluctuations were measured every quarter second on the beam dump during the irradiation, and the average of this current over every minute was used to calculate the average production rate of each individual nuclide during the irradiation. From these production rates, effective cross sections were determined for every nuclide using the measured beam current from the beam dump, which was calibrated for the divergence of the beam from the target position to the end of the beamline, and electron suppression using the suppressed Faraday cup placed at the target position prior to each irradiation, as previously noted. The target thicknesses that were measured following the aforementioned procedure were estimated to have a systematic error of 10%, since the alpha energy loss calculations rely on the best available range tables, which have a systematic error of 10%.

## Results and discussion

Table 1 contains all of the cross sections measured from the reactions of $^{147}$Sm($^{6}$Li,x) at 45 MeV, $^{148}$Sm($^{6}$Li,x) at 55 MeV, and $^{149}$Sm($^{6}$Li,x) at 55, 60, and 65 MeV beam energy. In addition to measuring many new ground state cross sections for all these unique reaction systems, cross sections for the metastable isomeric states of $^{148\textrm{m}}$Tb, $^{149\textrm{m}}$Tb, and $^{150\textrm{m}}$Tb are also reported for the first time. Since the available time to collect high statistics was low for the shorter-lived nuclides, some of the calculated errors for these nuclides are higher than for some of the other measurements.

**Table 1:** Measured cross sections for produced nuclides measured from the $^{147}$Sm($^{6}$Li,x) @ 45 MeV, $^{148}$Sm($^{6}$Li,x) @ 55 MeV, $^{149}$Sm($^{6}$Li,x) @ 55 MeV, $^{149}$Sm($^{6}$Li,x) @ 60 MeV, $^{149}$Sm($^{6}$Li,x) @ 65 MeV, reaction systems.

	${}^{147}\textrm{Sm}({}^{6}\textrm{Li},x)$ @ 45 MeV	${}^{148}\textrm{Sm}({}^{6}\textrm{Li},x)$ @ 55 MeV	${}^{149}\textrm{Sm}({}^{6}\textrm{Li},x)$ @ 55 MeV	${}^{149}\textrm{Sm}({}^{6}\textrm{Li},x)$ @ 60 MeV	${}^{149}\textrm{Sm}({}^{6}\textrm{Li},x)$ @ 65 MeV
Nuclide	$\sigma$ (mb)	$\sigma$ (mb)	$\sigma$ (mb)	$\sigma$ (mb)	$\sigma$ (mb)
$^{146}$Eu	66 ± 7	–	6.0 ± 0.6	33 ± 3	120 ± 10
$^{147}$Eu	220 ± 20	–	73 ± 7	140 ± 10	190 ± 20
$^{148}$Eu	47 ± 5	110 ± 10	170 ± 20	250 ± 30	350 ± 40
$^{150\textrm{m}}$Eu	–	–	19 ± 2	14 ± 2	–
$^{147}$Gd	0.83 ± 0.09	–	0.61 ± 0.09	1.1 ± 0.1	4.4 ± 0.6
$^{151}$Gd	–	–	73 ± 7	140 ± 10	210 ± 30
$^{148\textrm{g}}$Tb	17 ± 3	–	–	–	23 ± 2
$^{148\textrm{m}}$Tb	38 ± 6	17 ± 2	–	–	60 ± 9
$^{149\textrm{g}}$Tb	28 ± 3	20 ± 2	14 ± 2	32 ± 3	37 ± 4
$^{149\textrm{m}}$Tb	350 ± 50	240 ± 50	79 ± 14	390 ± 70	590 ± 80
$^{150\textrm{g}}$Tb	11 ± 1	–	66 ± 7	61 ± 7	47 ± 8
$^{150\textrm{m}}$Tb	83 ± 13	160 ± 20	560 ± 60	820 ± 100	600 ± 70
$^{151}$Tb	6.5 ± 0.9	13 ± 2	180 ± 20	140 ± 20	110 ± 10
$^{152}$Tb	–	–	21 ± 2	18 ± 2	–
$^{153}$Tb	1.4 ± 0.2	–	6.4 ± 0.8	6.0 ± 0.6	6.4 ± 1.1

**Figure 3:**
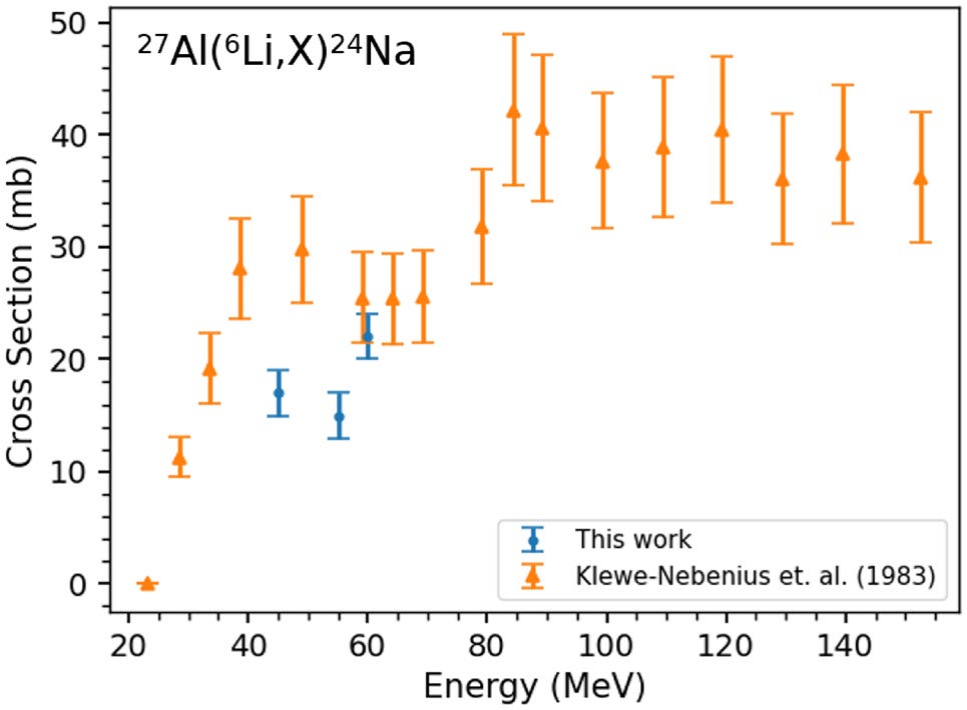
Cross sections for the $^{27}$Al($^{6}$Li,X)$^{24}$Na reaction compared with previous literature data by Klewe-Nebenius et. al.^34^.

We also measured cross sections for the $^{27}$Al($^{6}$Li,X)$^{24}$Na reaction on the Al backings of all the target foils. Since the Hyperion chamber is also primarily comprised of aluminum, only the data from the off-line, single-crystal detectors and not from Hyperion were used in these calculations to avoid background contamination from the other sources of activated aluminum. These cross sections can be compared with previous results in the literature performed by Klewe-Nebenius et. al.^34^, as a check for systematic error in our measurement and analysis. These cross sections are shown in Fig. 3 with comparisons to the literature. The cross section for $^{24}$Na at 65 MeV is not reported due to significantly higher activities of $^{24}$Na than expected (multiple orders of magnitude) resulting from the partial irradiation of the target frame. As the measurement of the beam current only records the beam that passes through the target and not the frame, this did not affect any of the target interactions. The value of the cross section in these measurements compared to the values presented by Klewe-Nebenius et. al.^34^ differs by about 10 mb in the most extreme case, which is within 2.5 standard deviations. This suggests systematic differences that are not understood between the two measurements. Additionally, the previous experimentalists in Reference 34 indicate that their lower energy measurements have“larger statistical errors” ^34^ than the 16% error reported for all of the results. The true cause of the discrepancy can at present only be hypothesized and could be tested with further measurements.

**Figure 4:**
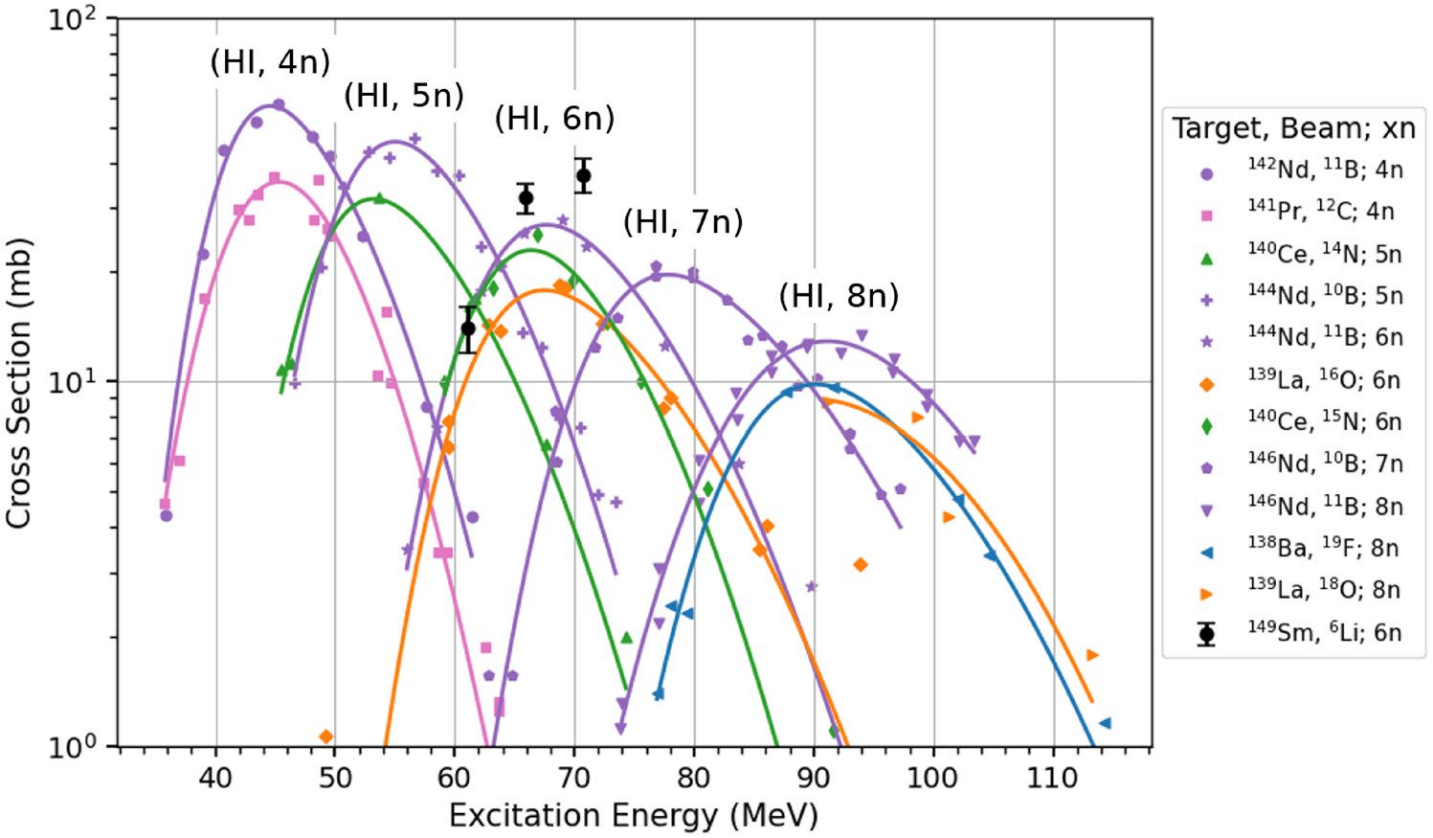
Cross sections for $^{149\textrm{g}}$Tb across a wide range of heavy ion reactions adapted from Alexander and Simonoff ^8^ with new cross sections measured by this work using $^{6}$Li beams on $^{149}$Sm shown by the star data points in black.

Since the cross sections reported by this work are the first of these reactions to be reported in the literature, only comparisons to similar reactions are possible. Alexander and Simonoff ^8^, for example, measured the production of $^{149\textrm{g}}$Tb across a range of heavy-ion production reactions, shown in Figure 1 of Ref. 8 and by the colored data in Fig. 4. For the $^{149}$Sm target, these cross sections are in agreement with the trends measured by Alexander and Simonoff ^8^. The measured results follow the same shape as exhibited by the other (HI,6n) curves, in the same excitation energy range. Additionally, the lighter projectile species result in higher cross sections for each of the measured reactions. As only one energy was measured for the cross sections on the $^{147}$Sm and $^{148}$Sm targets, these points were omitted for figure clarity since a trend with excitation energy is unable to be determined.

**Figure 5:**
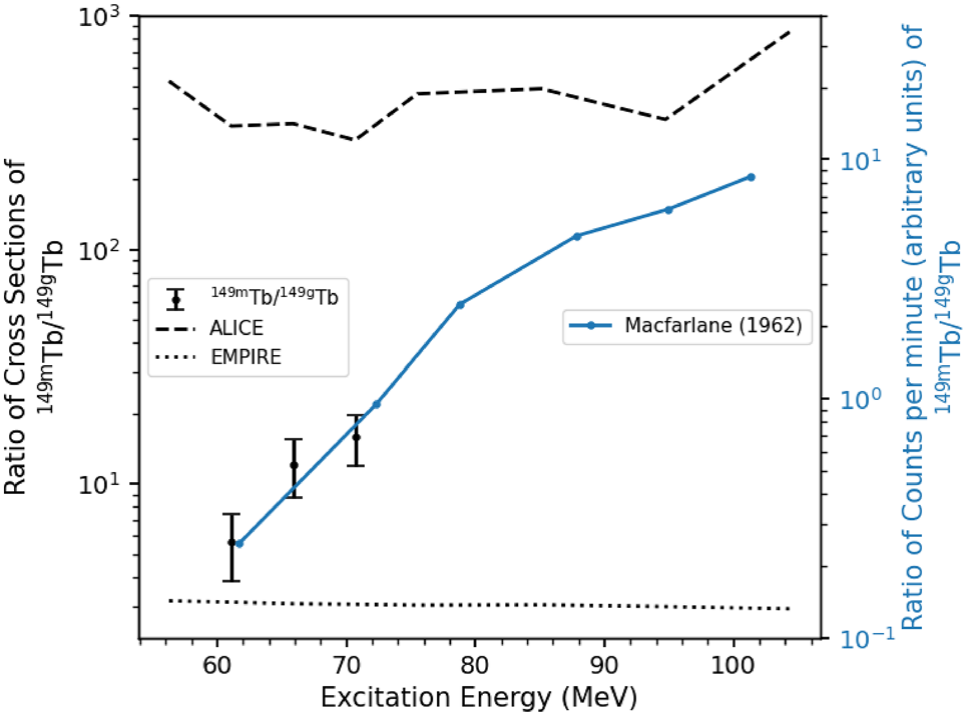
Comparison of the ratio of production of $\mathrm {^{149m}}$Tb to $\mathrm {^{149g}}$Tb between the measured cross sections from this work (black) and the data presented by Macfarlane ^17^ when $\mathrm {^{149m}}$Tb was first measured. ALICE and EMPIRE predictions of this ratio are shown by the dashed and dotted lines, respectively.

It is also of interest to compare the ratio of production of the ground state and metastable isomer of $^{149}$Tb to the previous measurements. The only measurement reported of this ratio was made by Macfarlane in 1962 using the $^{139}$La+$^{16}$O reaction ^17^. Figure 5 shows the comparison of Macfarlane’s data to this work. The results are only reported in arbitrary units of counts per minute rather than in mb or another comparable unit since alpha branching ratios for the state were not yet determined. The axes in Fig. 5 are proportional to each other but the proportionality constant is unknown. Therefore, the scaling of one axis relative to the other is arbitrary and is chosen to overlap the two data series well. Thus the agreement at a single energy is not meaningful, but the agreement at all energies simultaneously indicates that the two measurements are consistent.

Direct comparisons to the predictions made by the reaction codes ALICE and EMPIRE can also be made for this ratio. In these comparisons, ALICE predicts a ratio about 10$^{2}$ times higher than the measured results, while EMPIRE underpredicts this ratio by a factor of 2-5 depending on the excitation energy. This large overprediction by ALICE has been previously reported ^35^, and is due to the use of the Weisskopf-Ewing statistical model, which does not conserve angular momentum.

**Figure 6:**
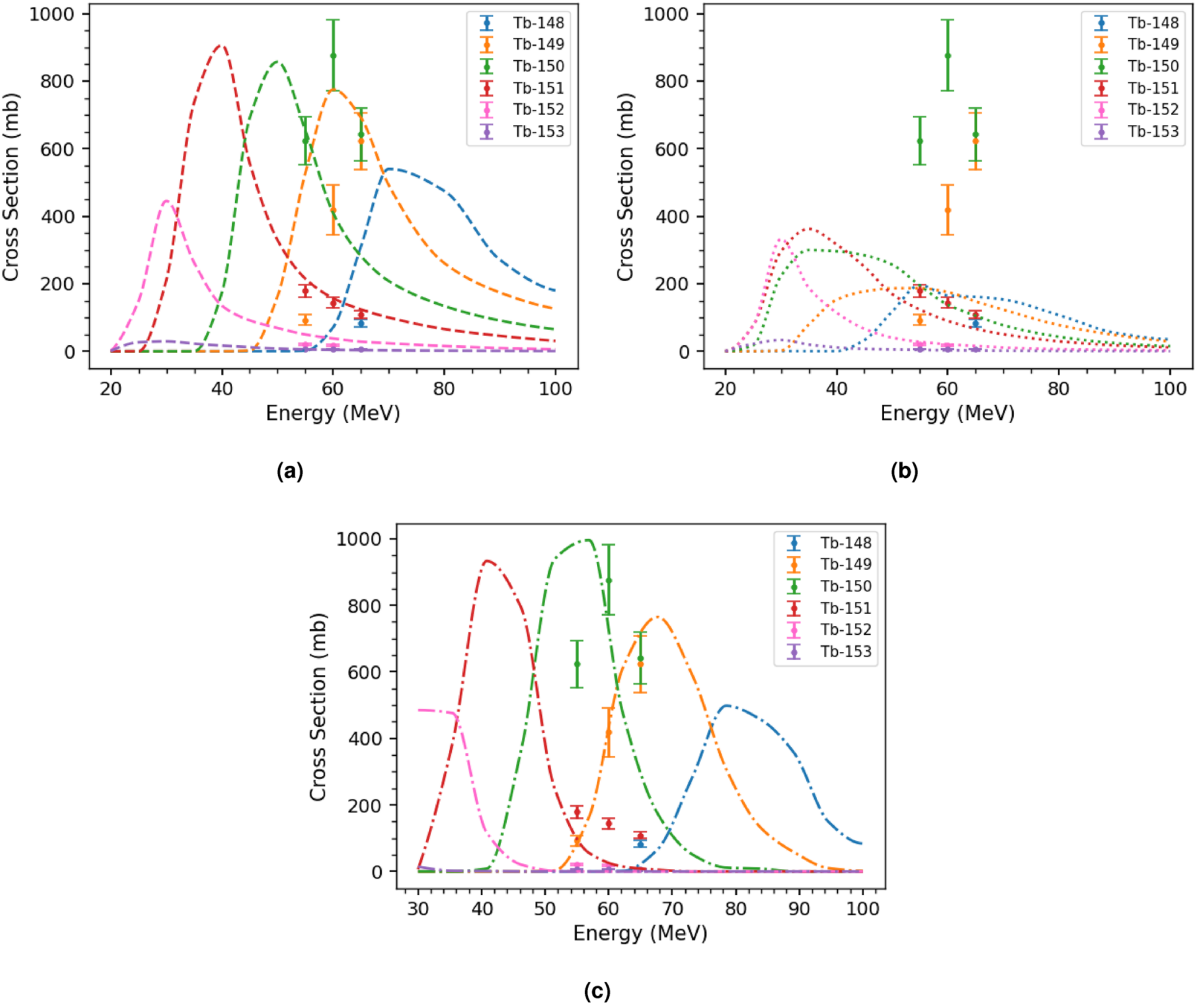
Comparison of measured total (ground state plus excited state) terbium cross sections to ALICE (**a**) , EMPIRE (**b**), and PACE4 (**c**) predictions for the $^{149}$Sm target systems.

Finally, the cross sections were compared with those predicted by the selected reaction codes. For comparison with PACE4, in addition to ALICE and EMPIRE, total cross sections (ground plus isomeric states) of the measured terbium isotopes were used, since PACE4 cannot distinguish between ground and isomeric states. These comparisons are shown in Fig. 6, for the cross sections measured and predicted for the $^{149}$Sm targets. From these comparisons, EMPIRE underpredicts the magnitudes of the cross sections for many of the Tb isotopes, although it agrees more closely with the data for the higher-mass isotopes. Both ALICE and PACE on the other hand, agree with the measured cross section magnitudes for all of the Tb isotopes reasonably well, although an energy shift in the predictions is required. The prediction made by ALICE for the peak of the $^{150}$Tb excitation function appears to be downshifted by about 10 MeV, while the same shift is only by about 5 MeV for PACE4. ALICE also predicts lower energies for the left side of the $^{149}$Tb excitation function by about 7 MeV, while the PACE4 predictions here agree with the measured data. The peak of the $^{149}$Tb excitation function and the projected peak of that for $^{150}$Tb have less of an energy separation from each other than predicted by either ALICE or PACE4. For the $^{147}$Sm and $^{148}$Sm targets, accuracy of the energy predictions is difficult to assess given that only a single energy was measured.

## Summary and conclusions

Cross sections have been measured and reported for 12 different nuclides for the $^{147}$Sm($^{6}$Li,x) at 45 MeV, $^{148}$Sm($^{6}$Li,x) at 55 MeV, and $^{149}$Sm($^{6}$Li,x) at 55, 60, and 65 MeV reaction systems. Cross sections for short-lived isomeric states of four of these nuclides were also measured, allowing for a complete picture of the nuclides produced and a more accurate comparison to predictive models that cannot distinguish between these isomers, such as PACE4. Of the three models tested, PACE4 most accurately predicts the energy peak of the excitation functions, for which the peak was measured, while both PACE4 and ALICE accurately predict the cross section magnitudes for the Tb isotopes. This work also presents comparisons with early heavy-ion measurements made by Alexander and Simonoff and Macfarlane that agree well with the data reported here.

Although the total $^{149}$Tb is produced with cross sections as high as 700 mb, the dominant form of its production is the metastable isomer which is not sufficiently long-lived, nor emits an alpha particle to be useful for TAT. Therefore, it would be most useful to explore other methods of production focusing on the production of the ground state isomer via light-ion acceleration.

## Supplementary Information

Supplementary Information.

## Data Availability

Data sets generated during the current study are available from the corresponding author on reasonable request.

## References

[1] McDevitt, M. R. et al. Radioimmunotherapy with alpha-emitting nuclides. *Eur. J. Nucl. Med.* **25**, 1341–1351 (1998). 9724387 10.1007/s002590050306

[2] Wilbur, D. S. Chemical and radiochemical considerations in radiolabeling with -emitting radionuclides. *Curr. Radiopharm.* **4**, 214–247 (2011). 22201710 10.2174/1874471011104030214

[3] Zalutsky, M. R. & Pruszynski, M. Astatine-211: Production and availability. *Curr. Radiopharm.* **4**, 177–185 (2011). 22201707 10.2174/1874471011104030177 PMC3503149

[4] Eychenne, R., Chérel, M., Haddad, F., Guérard, F. & Gestin, J.-F. Overview of the Most Promising Radionuclides for Targeted Alpha Therapy: The â€œHopeful Eightâ€. *Pharmaceutics* **13**, 906 (2021). 34207408 10.3390/pharmaceutics13060906 PMC8234975

[5] Zaitseva, N. G. et al. Terbium-149 for nuclear medicine. The production of Tb via heavy ions induced nuclear reactions. Czechoslovak Journal of Physics 53, A455–A458, 2003, 10.1007/s10582-003-0058-z.

[6] Wilkinson, J. T. et al. A heavy-ion production channel of Tb via Cu bombardment of Y. *Applied Radiation and Isotopes* **178**, 109935 (2021). 34555596 10.1016/j.apradiso.2021.109935

[7] Maiti, M., Lahiri, S. & Tomar, B. S. Investigation on the production and isolation of Tb from C irradiated natural praseodymium target. *Radiochim. Acta* **99**, 527–534. 10.1524/ract.2011.1839 (2011).

[8] Alexander, J. M. & Simonoff, G. N. Excitation Functions for Tb from Reactions between Complex Nuclei. *Phys. Rev.* **130**, 2383–2387. 10.1103/PhysRev.130.2383 (1963).

[9] Duchemin, C. et al. Production Cross-Section Measurements for Terbium Radionuclides of Medical Interest Produced in Tantalum Targets Irradiated by 0.3 to 1.7 GeV Protons and Corresponding Thick Target Yield Calculations. Frontiers in Medicine 8, 2021, 10.3389/fmed.2021.625561. 10.3389/fmed.2021.625561 PMC8149945 34055823

[10] Verhoeven, H. Tb radioisotopes for medical applications: Spallation cross section measurements and first isotope delivery from CERN-MEDICIS. Ph.D. thesis, KU Leuven (2018).

[11] Zagryadskii, V. A. et al. Measurement of Terbium Isotopes Yield in Irradiation of Eu Targets by He Nuclei. *Atomic Energy* **123**, 55–58. 10.1007/s10512-017-0299-8 (2017).

[12] Moiseeva, A. N. et al. Cross section measurements of Eu(He, 5n) reaction: New opportunities for medical alpha emitter Tb production. Scientific Reports 10, 2020, 10.1038/s41598-020-57436-6. 10.1038/s41598-020-57436-6 PMC6965643 31949230

[13] Kazakov, A. G., Aliev, R. A., Bodrov, A. Y., Priselkova, A. B. & Kalmykov, S. N. Separation of radioisotopes of terbium from a europium target irradiated by 27 MeV -particles. *Radiochim. Acta* **106**, 135–140. 10.1515/ract-2017-2777 (2018).

[14] Beyer, G. J. et al. Production routes of the alpha emitting Tb for medical application. *Radiochim. Acta* **90**, 247–252. 10.1524/ract.2002.90.5_2002.247 (2002).

[15] Steyn, G. et al. Cross sections of proton-induced reactions on Gd, Gd and Tb with emphasis on the production of selected Tb radionuclides. Nuclear Instrum. and Methods in Physics Research Section B: Beam Interactions with Materials and Atoms 319, 128–140, (2014) 10.1016/j.nimb.2013.11.013.

[16] Cavaier, R. F. et al. *Conference on the Application of Accelerators in Research and Industry, CAARI 2016, 30 October - 4 November 2016* (Ft, Worth, TX, USA, 2017).

[17] Macfarlane, R. D. Alpha-Emitting Isomeric State of Tb. *Phys. Rev.* **126**, 274–276. 10.1103/PhysRev.126.274 (1962).

[18] Tarasov, O. & Bazin, D. LISE++: Radioactive beam production with in-flight separators. Nuclear Instrum. and Methods in Physics Research Section B: Beam Interactions with Materials and Atoms 266, 4657–4664, 10.1016/j.nimb.2008.05.110(2008). Proceedings of the XVth International Conference on Electromagnetic Isotope Separators and Techniques Related to their Applications.

[19] Gavron, A. Statistical model calculations in heavy ion reactions. *Phys. Rev. C* **21**, 230–236. 10.1103/PhysRevC.21.230 (1980).

[20] Blann, M. New precompound decay model. *Phys. Rev. C* **54**, 1341–1349. 10.1103/PhysRevC.54.1341 (1996). 10.1103/physrevc.54.1341 9971470

[21] Herman, M. et al. EMPIRE: Nuclear Reaction Model Code System for Data Evaluation. *Nucl. Data Sheets* **108**, 2655 (2007).

[22] Guo, C. L. et al. Coupling effects on the fusion of at energies slightly above the Coulomb barrier. *Phys. Rev. C* **92**, 014615. 10.1103/PhysRevC.92.014615 (2015).

[23] Rath, P. K. et al. Complete fusion in Li+Sm reactions. *Phys. Rev. C* **88**, 044617. 10.1103/PhysRevC.88.044617 (2013).

[24] Rath, P. K. et al. Suppression of complete fusion in the Li +Sm reaction. *Phys. Rev. C* **79**, 051601. 10.1103/PhysRevC.79.051601 (2009).

[25] Rath, P. et al. Fusion of Li with Sm: Role of projectile breakup versus target deformation. *Nuclear Physics A* **874**, 14–31. 10.1016/j.nuclphysa.2011.10.004 (2012).

[26] Greene, J. P., Neubauer, J. & Deligiannis, D. A new evaporator system for target preparation at Argonne National Laboratory. Nuclear Instrum. and Methods in Physics Research Section A: Accelerators, Spectrometers, Detectors and Associated Equipment 561, 58–61, 10.1016/j.nima.2005.12.228(2006). Proceedings of the 22nd World Conference of the International Nuclear Target Development Society.

[27] Accelerators, Spectrometers, Hughes, R., et al. The Hyperion particle- detector array. Nuclear Instrum. and Methods in Physics Research Section A. Detectors and Associated Equipment 856, 47–52. 2017, 10.1016/j.nima.2017.03.012.

[28] mesytec GmbH. mvme - mesytec VME Data Acquisition. https://www.mesytec.com/downloads/mvme.html. Version 1.4.6.

[29] Bills, L. A. Measuring Production Pathways for the Targeted Alpha Therapy Candidate Terbium-149. Ph.D. thesis, Texas A &M University (2025). In press.

[30] Morrell, J. T. Curie: A python toolkit to aid in the analysis of experimental nuclear data. https://jtmorrell.github.io/curie/build/html/index.html (2019-). [Version 0.29; accessed April 26, 2024.].

[31] Brun, R. & Rademakers, F. ROOT - An Object Oriented Data Analysis Framework (1997).

[32] Ryan, C., Clayton, E., Griffin, W., Sie, S. & Cousens, D. SNIP, a statistics-sensitive background treatment for the quantitative analysis of PIXE spectra in geoscience applications. Nuclear Instrum. and Methods in Physics Research Section B: Beam Interactions with Materials and Atoms 34, 396–402, 1988, 10.1016/0168-583X(88)90063-8.

[33] “National Nuclear Data Center, B. N. L. NuDat (Nuclear Structure and Decay Data) (2008).

[34] Klewe-Nebenius, H., Michel, F., Monzel, H. & Neumann, B. Monitor Reactionsfor High Energetic Li-Projectiles. *Radiochim. Acta* **33**, 181–182. 10.1524/ract.1983.33.4.181 (1983).

[35] Fox, M. B. et al. Investigating high-energy proton-induced reactions on spherical nuclei: Implications for the preequilibrium exciton model. *Phys. Rev. C* **103**, 034601. 10.1103/PhysRevC.103.034601 (2021).

