# Supplementary material for: Cross sections of [image]Sm([image]Li,x) reactions for the production of [image]Tb for targeted alpha therapy
Source: Sci Rep. 2025 Nov 20;15:41057. doi: 10.1038/s41598-025-24894-9 (PMC12635120; doi:10.1038/s41598-025-24894-9)
Supplement: Supplementary file 1 — Supplementary Information. [file 41598_2025_24894_MOESM1_ESM.pdf]

Supplementary Information

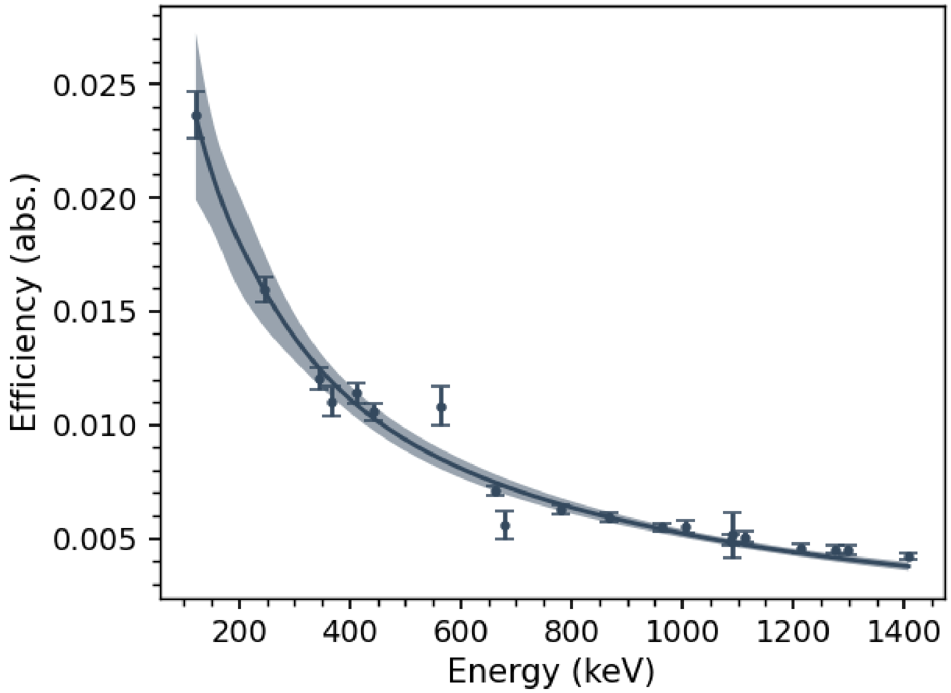

Supplementary Figure S1. Efficiency calibration curve with error band for the entire Hyperion detector array. Each of the points represents a measured γ-transition from the ^{152}Eu source.

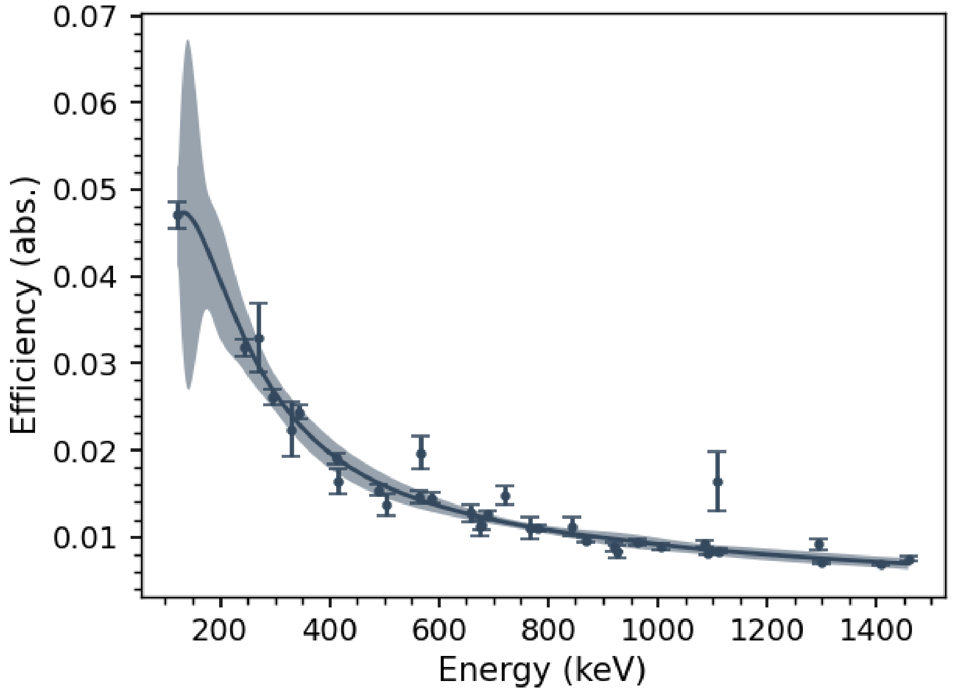

Supplementary Figure S2. Efficiency calibration curve with error band for one of the single crystal detectors (HPGe1). Each of the points represents a measured γ-transition from the ^{152}Eu source.

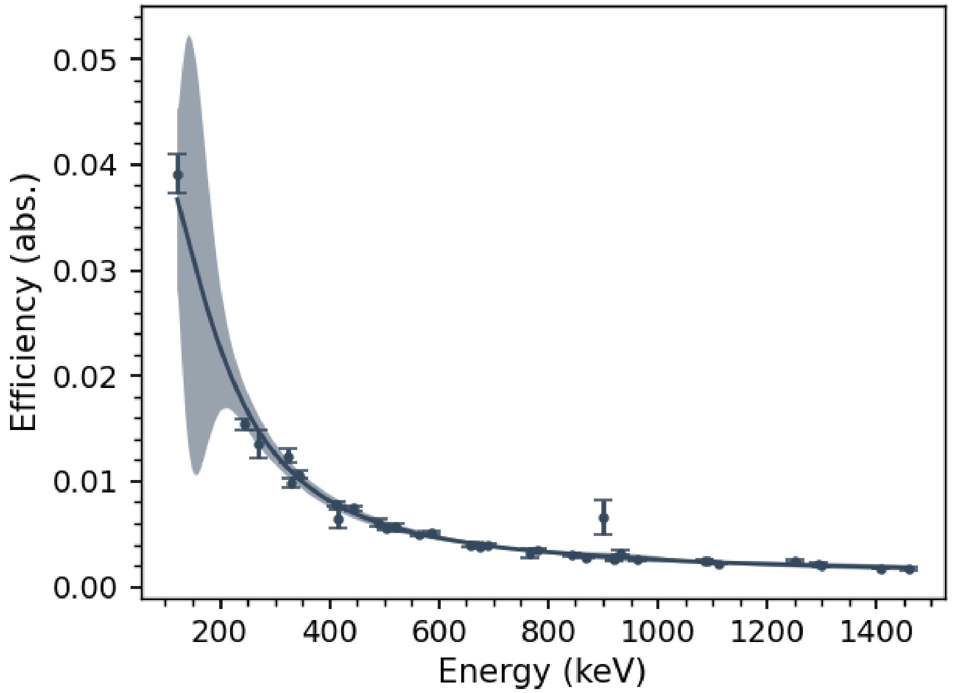

Supplementary Figure S3. Efficiency calibration curve with error band for one of the single crystal detectors (HPGe2). Each of the points represents a measured γ-transition from the ^{152}Eu source.
